# Federated Learning-Oriented Edge Computing Framework for the IIoT

**DOI:** 10.3390/s24134182

**Published:** 2024-06-27

**Authors:** Xianhui Liu, Xianghu Dong, Ning Jia, Weidong Zhao

**Affiliations:** CAD Research Center, Tongji University, Shanghai 201800, China; lxh@tongji.edu.com (X.L.); 1510501@tongji.edu.cn (N.J.); wd@tongji.edu.cn (W.Z.)

**Keywords:** industrial internet of things, edge computing, artificial intelligence, federated learning

## Abstract

With the maturity of artificial intelligence (AI) technology, applications of AI in edge computing will greatly promote the development of industrial technology. However, the existing studies on the edge computing framework for the Industrial Internet of Things (IIoT) still face several challenges, such as deep hardware and software coupling, diverse protocols, difficult deployment of AI models, insufficient computing capabilities of edge devices, and sensitivity to delay and energy consumption. To solve the above problems, this paper proposes a software-defined AI-oriented three-layer IIoT edge computing framework and presents the design and implementation of an AI-oriented edge computing system, aiming to support device access, enable the acceptance and deployment of AI models from the cloud, and allow the whole process from data acquisition to model training to be completed at the edge. In addition, this paper proposes a time series-based method for device selection and computation offloading in the federated learning process, which selectively offloads the tasks of inefficient nodes to the edge computing center to reduce the training delay and energy consumption. Finally, experiments carried out to verify the feasibility and effectiveness of the proposed method are reported. The model training time with the proposed method is generally 30% to 50% less than that with the random device selection method, and the training energy consumption under the proposed method is generally 35% to 55% less.

## 1. Introduction

With the advent of the information age and the proposal of the concept of intelligent manufacturing, the Industrial Internet of Things (IIoT) has become a popular focus of current research on both information technology and industrial technology. The Industrial Internet of Things (IIoT) refers to the concept of enhancing and optimizing industrial processes and applications using Internet and IoT technologies. It involves connecting sensors, devices, and other physical objects to the internet to enable data collection, monitoring, analysis, and automated control. This technology aims to improve production efficiency, reduce costs, and enhance the reliability and safety of industrial processes. IIoT finds wide applications across various sectors including manufacturing, energy, transportation, agriculture, and more, significantly transforming traditional industrial production methods [1]. By simulating human neural networks, artificial intelligence (AI) technology can play a role in various practical production application scenarios, including but not limited to image recognition, natural language processing, and decision support [2]. In the Internet of Things (IoT) context, various machine learning algorithms can make effective use of the large amounts of data generated by a large number of devices [3], while in the field of industrial production, AI can also make powerful contributions to tasks such as product defect detection, intelligent recognition and sorting, intelligent visual guidance and data analysis [4]. Thus, embedding AI technology into the IIoT is an important research direction in this field. Moreover, federated learning, as a method for training AI models in distributed systems, can effectively address the challenges that arise when training AI models in the IIoT [5].

In recent years, cloud-based computing has emerged as an open platform for the training and deployment of IIoT AI models by virtue of its dynamic expansion capabilities, flexible deployment capabilities, low cost and high efficiency [6]. However, the cloud computing scheme has the two following problems. First, in the process of training and applying reasoning models in the cloud, there will inevitably be a delay in data transmission, which presents a major challenge for time-sensitive IIoT applications [7]. Second, some enterprises have high requirements for data privacy and security, and cloud computing solutions inevitably face problems of data security [8]. Therefore, to overcome these problems of transmission delay and data security, the edge computing scheme has been proposed [9]. In the IIoT application scenario, edge computing can effectively alleviate the above shortcomings of cloud computing. Overall, the relationship among edge computing, cloud computing, and federated learning can be summarized as follows. Edge computing handles and preprocesses data, reducing the need to transmit data to the cloud, thus saving bandwidth and reducing latency. Cloud computing complements edge computing by providing storage and computational resources, supporting large-scale data processing and analysis. Federated learning leverages interactions between edge devices and center servers to enable collaborative learning across distributed data sources, thereby enhancing global model performance. However, existing AI-oriented edge computing frameworks generally have the following problems, and our research motivation is to provide a general solution to the following problems and limitations, as well as methods and frameworks for the rapid deployment of AI in the IIoT and the selection of federated hierarchical learning devices in energy computation and time-delay-sensitive scenarios. First, the high coupling between hardware and software in IIoT edge devices poses a challenge to the existing frameworks. The existing edge computing frameworks need a software-defined edge computing architecture that does not rely on specific hardware architectures. Second, the deployment and delivery methods of AI models, as well as their interactions with the device side, are complex steps in the context of IIoT applications. Third, the computing power of IIoT devices is generally insufficient, meaning that the timely and effective completion of the training of local models cannot be ensured, potentially making it difficult to meet real-time industrial needs. Fourth, IIoT applications are typically highly sensitive to issues of delay and energy consumption, and further study is needed on how to reduce delays and computing resource consumption in the federated learning process.

To address the above challenges, this paper proposes a federated learning-oriented edge computing framework for the IIoT. First, we propose an edge computing framework in which edge computing nodes and devices closely cooperate for access and interaction. By virtue of the software-defined nature of this framework, the hardware and software of the edge gateway nodes are decoupled, and the access protocols of the devices do not depend on the specific device type or the device architecture of the edge computing nodes. This framework can support the rapid delivery and deployment of AI models. On the basis of this framework, we present the design and implementation of an edge computing system that supports the realization of the whole process of data collection from the device side to the edge and AI model deployment from the cloud to the edge. Then, we propose a time series-based device selection and computation offloading method for use in the federated learning process. This method enables the purposeful selection of edge computing nodes and the partial offloading of computing tasks from the device side to the edge to address the resource allocation problem in the presence of an edge computing center. Through this method, the delay and energy consumption of the system in the federated learning process are optimized. Finally, we report experiments conducted to verify the feasibility of the proposed architecture and the superiority of the proposed method in terms of delay and energy consumption for the case of training a multilayer perceptron (MLP) network on the MNIST dataset.

The contributions of this paper are as follows:

(1) A software-defined AI-oriented three-layer IIoT edge computing framework is proposed to overcome the challenges of deep hardware and software coupling, diverse access protocols, and difficult deployment of AI models in the IIoT.
(2) An AI-oriented edge computing system based on a microservice architecture is designed and implemented. This system supports the realization of the whole process from device-to-edge data collection and processing to AI model distribution and deployment.
(3) A time series-based method of device selection and computation offloading for use in the federated learning process is proposed for IIoT edge computing to reduce both training time delay and energy consumption, thereby reducing long-term costs.
(4) Experiments are designed and implemented to evaluate the proposed method. Through a series of experimental analyses, we verify the feasibility and superiority of the proposed method.

The rest of the paper is organized as follows. The related works are reviewed in Section 2. In Section 3, the details of the proposed scheme and algorithm are provided. The results of the experiment of the proposed scheme and algorithm are discussed in Section 4. Section 5 discusses the superiority, in terms of time consumption and energy consumption, and limitations of the proposed scheme.

## 2. Related Research

This section summarizes the research on the IIoT applications of edge computing and AI models.

### 2.1. Edge Computing Architecture for the IIoT

IIoT edge computing was proposed to solve the problems of data security and transmission delay faced by cloud computing in industrial production. Sha et al. (2020) [10] proposed a generic edge-centric IoT architecture, explaining how the edge layer interacts with IoT application users, the cloud, and IoT end devices. Sodhro et al. (2020) [11] proposed an AI-based edge computing platform architecture that consists of adaptable edge nodes, adaptable network nodes and adaptable application nodes. The edge nodes are responsible for collecting and analyzing data using AI algorithms, the network nodes are responsible for obtaining node information and for transmission through the network, and the application nodes are responsible for running IIoT applications, such as real-time monitoring and error diagnosis. Zhao et al. (2022) [12] proposed an edge computing network using digital twin technology. With the help of digital twins, this edge computing network can connect IIoT devices more efficiently. Mai et al. (2021) [13] considered the limited computing resources of mobile edge computing and discussed a method of decomposing and distributing the execution of critical tasks among network devices.

Nguyen et al. (2021) [14] applied AI to model distributed networks to improve transmission efficiency. Mwase et al. (2022) [15] proposed a new AI strategy based on distributed machine learning (DML), which involves training AI models completely at the edge, and discussed optimizations to reduce both the size of the training data to be transmitted and the transmission scale, satisfying the needs of edge deployment and focusing on the processing of low-performance devices. Torres-Charles et al. (2022) [16] focused on collaborative sharing in the cloud at the edge and proposed a new cloud–edge computing architecture layout from the perspective of cloud–edge integration. Kok et al. (2022) [17] proposed an edge computing framework with the help of AI. They used AI algorithms to solve the communication, computing, caching and control (4C) problems in an IoT network and defined the network model and related mathematical formulas under their framework. In accordance with the challenges faced by different edge computing application scenarios, corresponding potential and feasible AI solutions were given. Zhao et al. (2023) [18] focused on the flexibility, security and real-time performance of the IIoT framework, proposed a new three-layer software-defined IIoT control architecture, and proposed the use of decentralized control devices (DCDs) as device execution units to control industrial devices and perform IIoT tasks. In this paper, we study an application scheduling algorithm for use in time-sensitive scenarios and propose a computation-based decentralized network intelligent IIoT application deployment (DNAI2) problem and its solution. Kumar et al. (2023) [19] studied the problem of multidimensional data processing in the cloud–edge framework of the IIoT and provided a method for processing IIoT data at the edge. Zhang et al. (2023) [20] considered the trust problem among complex heterogeneous devices in the IIoT. The aforementioned studies have examined industrial IoT edge computing architectures from various perspectives, with a common focus on transmission rates of network models, network nodes at edge computing centers, or optimized deployment of applications on edge devices.

### 2.2. Training AI Models in the IIoT and Federated Learning 

Studies on the applications of AI models in the IIoT focus on the efficiency and security of the AI model application process [21] and methods for training and deploying AI deep learning models in IIoT architectures. These methods include cloud training [22], edge training [23], cloud–edge collaborative training [24], and smart device-end model training [25].

Bellavista et al. (2020) [26] proposed a three-layer computing architecture in which on-site, edge, and cloud resources are used to run AI models in a collaborative manner. In this architecture, on-site data are input into an AI model, and model training is performed at the edge. The trained AI model is then transmitted to the cloud, and the cloud distributes it to each edge server that needs to use the AI inference model. Sun et al. (2020) [27] also proposed an AI computing framework based on the IIoT. In this architecture, edge servers and cloud servers work together to provide services for IIoT AI applications. AI models are trained in the cloud, and the trained AI reasoning models are deployed at the edge to perform inference on actual data. McClellan et al. (2020) [28] proposed a method of applying deep learning in mobile edge computing (MEC) using 5G technology. With the fast data transmission of 5G networks, deep learning models can be run at the mobile edge, and the data can be stored. 

Federated learning was initially proposed by Google in 2016 as a privacy-preserving distributed machine learning paradigm [29]. When integrated with cloud computing and edge computing, federated learning efficiently utilizes the computational power of dispersed terminal devices for parallel computation. It minimizes intermediate results synchronization to enhance the efficiency of distributed machine learning [30]. Below are some recent studies on federated learning focusing on model performance and privacy protection. Jiang et al. (2024) [31] integrated federated learning and split learning into satellite-terrestrial integrated networks (STINs), introducing advanced frameworks such as split-then-federated learning and FedSL-LSTM. Their approach addresses privacy and efficiency concerns in B5G/6G mobile communication, demonstrating superior performance in electricity theft detection. Parra-Ullauri et al. (2024) [32] introduced kubeFlower, a Kubernetes (K8s) operator for federated learning. It ensures privacy through secure resource isolation and integrates differential privacy via P3-VC. Tested on both cloud and edge nodes, their approach showcases robust privacy preservation in federated learning environments. Mhaisen et al. (2022) [33] studied a hierarchical federated learning system with edge training to optimize the selection problem of edge users. With the help of this system, AI models can achieve faster convergence and better accuracy during the training process. Baccour et al. (2022) [34] proposed deploying a federated learning training framework, a decentralized reinforcement learning training framework, and an active learning training framework in a decentralized network and studied algorithm models based on the above three frameworks. Moreover, the methods of model inference in a decentralized network for corresponding models trained based on the above three frameworks were studied while considering the privacy and security of the data during the transmission process. Salim et al. (2023) [35] proposed a computational framework based on information fusion. With the help of this framework, the training of artificial neural network models based on federated learning will use fewer training rounds, thereby reducing the consumption of computing resources and the training time cost while improving the model accuracy. Phan et al. (2023) [36] proposed an IIoT edge framework based on blockchain and federated learning. Within this framework, the federated learning scheme inherits fully homomorphic encryption and splitting-based privacy, making it more conducive to protecting data privacy and security when building AI models for IIoT networks.

However, due to constraints such as network communication, local computation costs, and device uptime, aggregation servers can only select a limited number of clients to participate in each training round [37]. Therefore, a core process in federated learning protocols is the “client selection” before each training round begins. Several widely applied federated optimization algorithms, including FedAvg [29], FedProx [38], and FedYoGi [39], employ random client selection algorithms. Some methods have improved upon client selection. For instance, DCS [40] minimizes communication costs by filtering clients based on expected value thresholds. However, it calculates the local update model value using a global validation set, potentially leading to information loss for models of lower value not participating in global aggregation rounds. FedCS [41] addresses the maximization of device selection by transforming it into a submodular maximization problem under knapsack constraints using greedy algorithms based on local model updates and transmission times. However, it does not necessarily minimize latency or energy consumption and lacks fairness considerations in device selection. Similarly, FedMCCS [42] converts the maximization of device selection into a double-layer maximization optimization problem under knapsack constraints, encountering similar issues. FedCCPS [43] aims to minimize overall training time by sorting local update times using K-means clustering and binary partitioning. However, it does not consider scenarios with constrained energy consumption.

## 3. Materials and Methods

This section first proposes a software-defined AI-oriented three-layer IIoT edge computing framework in Section 3.1. In the device layer, container virtualization technology is used to solve the problems of deep coupling between the hardware and software of devices and protocol diversity. In the data layer, the data and virtual device models are stored, and this layer is also responsible for the forwarding of device data. In the AI model layer, the steps of AI model application are described to solve the difficult problem of AI model deployment. Then, on the basis of this edge computing framework, we design and implement an AI-oriented edge computing system based on the microservice architecture in Section 3.2 and describe the functional modules of the system in the protocol service layer, data layer and application layer. Finally, we analyze the time and energy consumption costs of the federated learning system in Section 3.3 and propose a time series-based method of device selection and computation offloading to solve the problems of the general insufficiency of the AI computing power at the edge in the IIoT and the sensitivity to system delay and energy consumption.

### 3.1. Software-Defined AI-Oriented Three-Layer IIoT Edge Computing Framework

**Figure 1:**
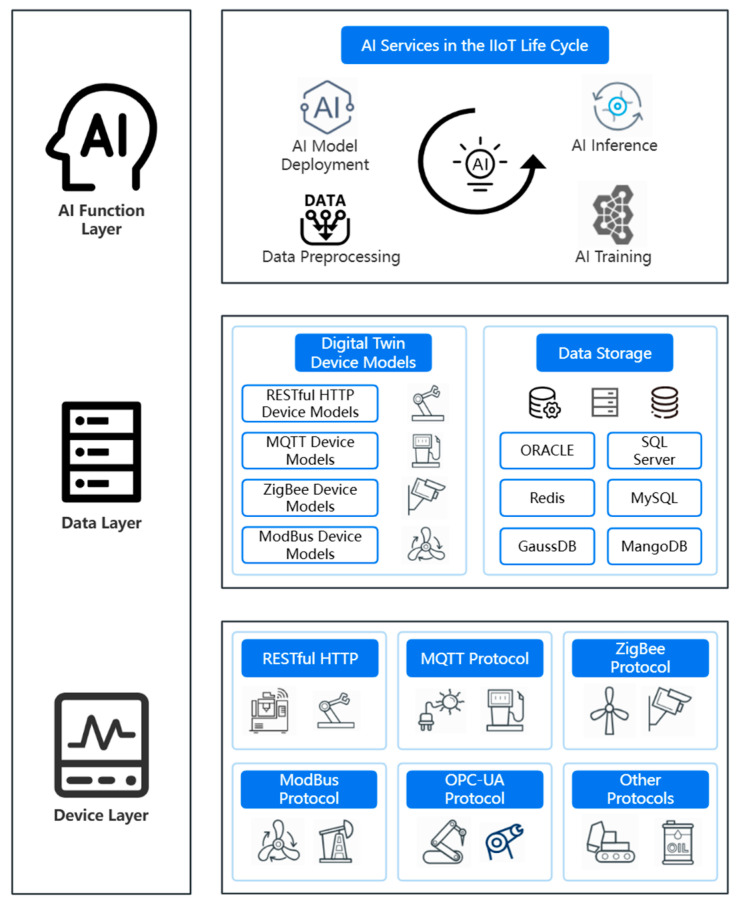
Software-defined AI-oriented three-layer IIoT edge computing framework.

This section proposes a software-defined, AI-oriented, three-layer IIoT edge computing framework, which is used to realize all necessary functionalities for edge computing, from device access to AI model training and inference. The overall framework is divided into three layers, as shown in Figure 1. From the bottom up, these layers are the device layer, the data layer and the AI function layer.

#### 3.1.1. Device Layer

Devices in the IIoT device layer have the characteristics of deep coupling between software and hardware, strong heterogeneity, and different access protocols [44]. In addition, in the traditional operational technology (OT) commonly used in industry, there is still no method available to support the rapid deployment of information technology applications [45]. The existing common IIoT device access protocols include RESTful HTTP, MQTT, ZigBee, ModBus, and OPC-UA. Among them, OPC-UA, as an enabling technology for industrial modeling, can describe device properties and functions in the form of object models for various industrial devices and provide standard programming interfaces and standard communication protocols [46]. However, many existing devices do not support this emerging protocol. Another software-defined device access method is virtualization technology [47].

By means of virtualization technology, the processes of data acquisition, protocol parsing and data processing can be encapsulated into a single independent device communication protocol image via container virtualization [48]. Data acquisition: through edge computing devices (such as sensors, programmable logic controllers, remote terminal units, industrial computers, etc.), data are collected at the production site and processed in real time, and the collected data are transmitted to the edge for further analysis and processing. Protocol parsing: the raw data collected from the edge devices are parsed and converted into a standardized data format in accordance with the corresponding protocol mirror to facilitate subsequent storage, analysis and application. Data processing: by using a rule engine and a hierarchical control method, the data collected and parsed in the protocol image are processed and analyzed, and local tasks are processed below the edge device connection end, reducing the dependence on the edge and the cloud and improving the real-time performance and efficiency of data processing while ensuring the security and integrity of the data.

At the software level, the IIoT device layer realizes isolation between device access programs and general industrial applications. In this way, more flexible deployment and container migration capabilities can be supported. In addition, to better meet the real-time performance requirements of device access, time-sensitive software-defined networking (TSSDN) is also adopted. In this networking paradigm, time-sensitive networking is implemented on general SDN switches, which can theoretically eliminate transmission jitter [49]. By integrating the data acquisition, protocol parsing and data processing flows of various common protocols through container virtualization, the industrial Internet platform can realize the efficient collection and processing of production data and provide strong support for increasing the digitalization, intelligence and efficiency of the production process.

#### 3.1.2. Data Layer

The data layer includes digital twins of various devices [50] and data storage. On the one hand, the data layer performs virtual device mapping to the digital space for the real physical devices. Each virtual device periodically checks the state of the corresponding real physical device, describes the functions of the physical device, and collects information on the available computing and network resources of the real physical device. The storage of virtual devices is divided in the protocol dimension, where the corresponding models include RESTful HTTP device models, MQTT device models, ZigBee device models, ModBus device models, etc. On the other hand, the data layer is responsible for the collection, storage and forwarding of device data. The databases required for data storage include all kinds of relational databases and nonrelational databases as well as distributed databases. Facing the device side, the southbound interface of the data layer is responsible for communicating with the device layer to complete all operations related to the device life cycle, such as device module modeling, discovery, monitoring, destruction, backup, and migration. Facing the AI server, the northbound interfaces of the data layer provide abstract data types and network programming interfaces for AI applications to achieve network awareness and control capabilities. In Section 3.3, we design a time series-based method of device selection and computation offloading for the forwarding of device data.

#### 3.1.3. AI Function Layer

The AI model application process can be divided into four main steps: training model deployment, data collection and preprocessing, model training, and inference model deployment. The deployment of AI models is designed in a data-driven manner. For the selection and deployment of AI models for the data collected from the data layer, including structured data, unstructured data and semistructured data, APIs and various other components are integrated based on industrial application modeling, facilitating the rapid development of IIoT AI applications. Field engineers can quickly design and deploy industrial AI applications for the training and application of models for reasoning on real data without worrying about specific implementation details or complicated deployment steps. The dynamically collected data are obtained from the device layer, are then loaded and extracted through real-time stream calculations, and are finally stored in the data layer in either a central or distributed manner. The AI function layer accesses these massive-scale data through a standard Open Database Interconnection (ODBC) interface. For the AI model service, Docker technology is used to select basic images such as TensorFlow Serving, and the compilation environments of C++, Python, Go and other languages are adapted and integrated with JupyterLab. The model framework and dependencies are installed by means of rule chains and WebSocket to construct an integrated AI service development environment supporting multiple types of programming languages. Accordingly, model loading, training, and reasoning scripts can be configured by field engineers, and AI applications can be rapidly developed once the environmental variables have been configured. In addition, the web version provides a graphical development environment based on TensorBoard, ECharts and other technologies to realize the configuration operation and atomic interaction of the metamodel and to support the rapid construction of intelligent models in a graphical way.

### 3.2. AI-Oriented Edge Computing System

**Figure 2:**
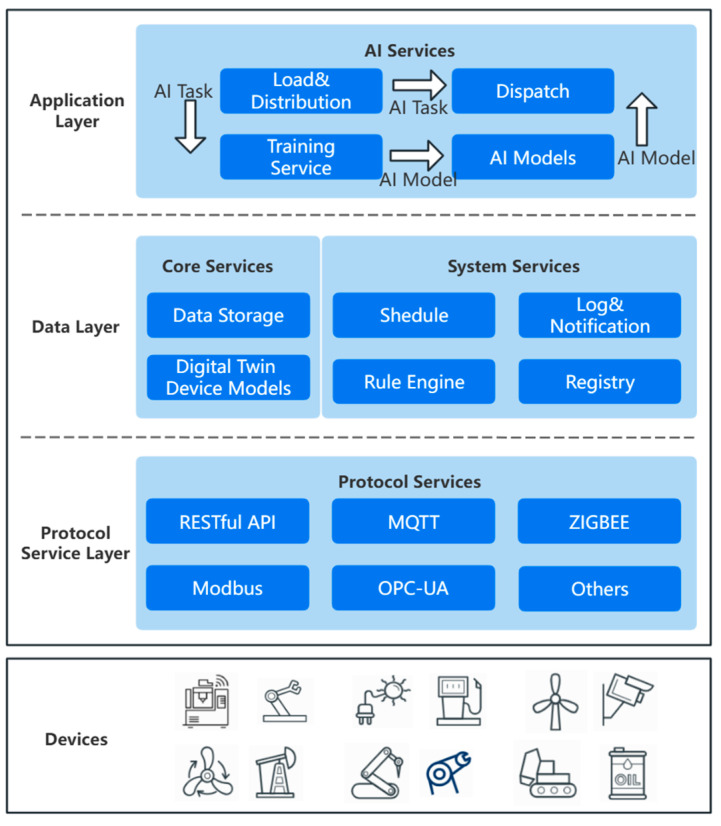
Architecture diagram of the AI-oriented edge computing system.

Based on the above software-defined AI-oriented three-layer IIoT edge computing framework, we present the design and implementation of an AI-oriented edge computing system in this section. The system architecture is shown in Figure 2. In this edge computing system, a microservice architecture [51] is adopted. There are three layers, namely, the protocol service layer, the data layer, and the application layer, corresponding to the access part of the device service layer, the data layer and the AI function layer, respectively, in the above software-defined AI-oriented three-layer IIoT edge computing framework. The functions of the detailed service modules in each layer are listed hierarchically below.

#### 3.2.1. Protocol Service Layer

The protocol service layer is the lowest layer of the entire edge computing architecture and is responsible for direct interaction with the underlying devices. The communication protocol between the protocol service layer and the underlying devices is deployed in the form of device services using container technology. The interaction modes include built-in RESTful API, MQTT, ZIGBEE, Modbus, and OPC-UA modes, among others.

When a specific device needs to be connected to the edge system, the registration and configuration function in the function layer will select the corresponding device service in accordance with the communication protocol used by the device and register the device information in the device service list to complete device access. This protocol service can be rewritten and extended. There are many device access protocols for the IIoT. To expand support for a variety of different device access protocols, a variety of new and different device protocols can be designed in container form and deployed in the protocol service layer.

#### 3.2.2. Data Layer

The core data service functions reside in the data layer. The Digital Twin Device Models module corresponds to the digital twins of devices, which store the configuration data of the corresponding virtual container devices, including the data required for IIoT device configuration and the data for pairing virtual devices with device services. The communication between the system and each specific device needs to comply with a specific communication protocol, and data are transmitted in a specific format based on a specific configuration. These configuration data are stored in this digital twin module. When a device service is connected to a device through the device service layer, the device’s configuration information and function API are registered in this module. When a device needs to operate or communicate, the relevant configuration information can be obtained from this module.

The Data Storage module is responsible for collecting device data, storing the data transmitted by the device service layer, and performing related simple processing and management of the data. All the data transmitted by the device service layer to the functional layer are received and stored by the Data Storage module. The databases used for data storage can include relational databases such as SQL Server, MySQL, and Oracle databases or nonrelational databases such as Redis and MongoDB databases. In actual industrial production scenarios, field engineers can decide which key data should be stored and can simply process the data. The data stored in the Data Storage module can be accessed by other microservices in the microservice group that have access permissions, thereby enabling data storage and data interaction between IIoT applications.

The Registry module is responsible for registering and configuring other microservice applications. It is the registration and configuration server in a microservice group. After each microservice function is started, the Registry function will register the configuration properties of the microservice in the console through a RESTful API. All data are stored in the form of key–value pairs, and when one microservice server communicates with another microservice server, the relevant configuration information will also be obtained from the Registry module.

The Log&Notification function handles system notifications and logging. In the Schedule and Rule Engine modules, the scheduling and rule engine policies can be customized by engineer users. The Rule Engine module provides invocation methods to support different microservice scheduling strategies and data.

#### 3.2.3. Application Layer

The application layer of the AI-oriented edge computing architecture is designed to support a generally applicable AI model training cycle at the edge. This cycle includes deployment, loading and distribution; training services; storage functions for AI models; and distribution functions for computation offloading and uploading model updates.

Load&Distribution and Dispatch services: The Load&Distribution service is responsible for loading and scheduling AI tasks. The cloud platform deploys AI tasks to the edge computing platform through the Dispatch service, and once these tasks are received, they are loaded and scheduled by means of the Load&Distribution service. Specifically, when the Load&Distribution service receives a request to issue AI tasks, it parses the tasks and determines whether it needs to use other mutually trusted nodes in accordance with the current remaining computing resources. If the local edge computing resources are insufficient to support all the AI tasks, then some tasks need to be decomposed and sent back to Dispatch. Dispatch transfers the tasks that need computational offloading to other mutually trusted nodes or sends models that have been trained in the local edge framework back to the cloud.

Training Service and AI Models: The Training Service is responsible for the real-time training of AI models running on edge devices in the field. AI tasks, including configuration information and AI models, are received by the application layer of the edge computing architecture and transmitted to the Training Service for local fusion and real-time training. The training results, namely, the AI models, are subsequently sent back for storage and further transmission.

### 3.3. Time Series-Based Method of Device Selection and Computation Offloading

In this section, an energy queue-based device selection and computation offloading method is proposed for use in federated edge learning. In general, in federated edge learning, the devices used for training local models are generally selected randomly [52]. However, this random device selection approach does not fully consider the problems of delay and energy consumption in the IIoT application scenario. Therefore, an energy queue-based device selection and computation offloading method is proposed in this section. By maintaining a time series consisting of the estimated times needed for one round of local model training on each edge device, the devices for the current round of training can be selected from the near range of the queue, and the part of the calculation whose energy consumption would exceed the maximum value among the devices in each round is offloaded to the edge computing center for completion. In this way, the energy consumption and time cost of the federated edge learning system can be reduced in the long term.

#### 3.3.1. Federated Edge Learning

**Figure 3:**
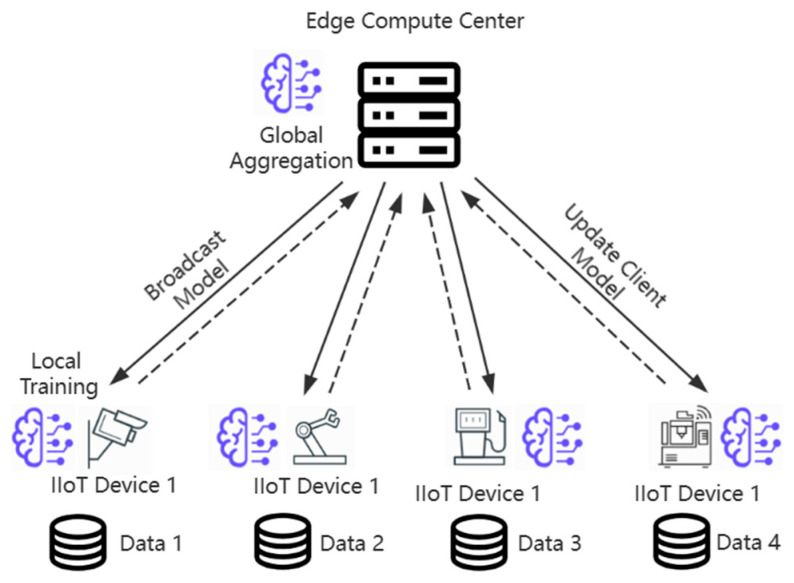
Training process under the federated learning model.

Figure 3 shows the federated learning model training graph in the system. There are N devices in the system, and k of them are selected for training in each round. The local models at the edge have their own data sets generated from the field in real time, they train the data in their own scope, and then upload the trained model parameters to the edge computing center, which then performs model fusion. Subsequently, the fused model is distributed back to the devices, which fuse it with their own trained models to obtain new local models and perform iterative training again.

The mathematical formulas describing the process of federated learning and the time and energy consumption calculations are given below.

In each round, the edge compute center selects a set of devices, denoted as $K^{t}$, to participate in model training. $M^{t}$ represents the global model parameters at the beginning of a round of training, $M_{i}^{t}$ represents the parameters of the local model before one round of training, $M_{i}^{t+1}$ represents the parameters of the local model after one round of training, and $L\left(M_{i}^{t}\right)$ represents the loss function for local model training. Subsequently, the edge compute center sends the latest global model $M^{t}$ to all selected devices:

(1) $$M_{i}^{t}=M^{t},\;i\in K^{t}$$

The selected devices update their local models using the received global model and their own datasets, employing the stochastic gradient descent algorithm, where δ denotes the step size:

(2) $$M_{i}^{t+1}=M_{i}^{t}-\delta \nabla L\left(M_{i}^{t}\right)$$

The global model parameters at the end of a round of training are calculated through weighted averaging of the local model parameters in accordance with the relative data proportions:

(3) $$M^{t+1}=\sum_{i=1}^{k}\frac{D_{i}}{D}M_{i}^{t+1}$$

where D is the total amount of data used in this round of training and $D_{i}$ is the size of the local dataset of device ⅈ.

The time consumption for each local training is

(4) $$T_{i}=\varepsilon \gamma D_{i}\frac{1}{f_{i}}$$

where ε represents the number of iterations; γ represents the average number of CPU clock cycles to compute a unit amount of data, which is regarded as a constant for a given calculation task; and $f_{i}$ represents the CPU frequency of the device for the calculation task. The calculation frequencies of different devices are different, but the frequency of a single device remains consistent throughout a calculation task. It must be noted that in the above equation, we have simplified the calculation of local training time consumption to a certain extent. Strictly speaking, the number of data points in local computation and training time do not necessarily exhibit a strictly linear relationship. For example, support vector machines (SVM) exhibit quadratic complexity, while methods like random forests, decision trees, convolutional neural networks (CNN), and multilayer perceptrons (MLP) have quasilinear complexity [53,54,55]. For certain methods in the IIoT, such as MLP used in our experimental section, the size of the dataset and training time can be approximated to be linearly related. The approximate calculation of energy consumption in the following equations follows a similar logic.

The energy consumption for each local training is approximated as

(5) $$E_{i}=\rho \varepsilon \gamma D_{i}f_{i}^{2}$$

where ρ is a capacitance coefficient constant, which represents the energy consumption of the CPU computing module. The energy consumption is affected by the working voltages of the electronic components, and it is not easy to calculate the exact value; therefore, we use an approximate value.

The total time consumption for the calculation is

(6) $$T_{comp}=\max{\left\{T_{i}\right\}}_{i=1}^{k}+T_{0}$$

where $T_{0}$ represents the time needed for device selection, model parameter transmission and model fusion at the edge computing center, which is generally small compared with the local model training time and thus can be ignored. Therefore, the above expression simplifies to

(7) $$T_{comp}=\max{\left\{T_{i}\right\}}_{i=1}^{k}$$

(8) $$T_{comp}=max{\left\{D_{i}\frac{1}{f_{i}}\right\}}_{i=1}^{k}\cdot \varepsilon \gamma$$

The total energy consumption is calculated as

(9) $$E_{comp}=\sum_{i=1}^{k}E_{i}$$

(10) $$E_{comp}=\sum_{i=1}^{k}{\rho \varepsilon \gamma D_{i}f_{i}^{2}}_{i}$$

The optimization goal is to minimize $T_{comp}$ while ensuring that $E_{comp}$ does not exceed $E_{0}$, which represents the threshold for the total energy consumption of the entire device system.

#### 3.3.2. Time Series-Based Method of Device Selection and Computation Offloading

To reduce the total training time and energy consumption, a time series-based device selection and computation offloading strategy is now considered for the local computing tasks. Computational offloading refers to transferring part of the computing task burden of an edge device, i.e., part of the dataset, to the edge computing center when the computing capacity of the edge device is insufficient; then, the edge computing center performs the calculation on behalf of the edge device.

According to Equation (8), the computation time of the system is related to the longest time consumption among the devices selected in each round. Therefore, we maintain a time series representing the lengths of time needed for training on each device and try to select devices with similar time consumption for training during each round of device selection; in this way, the total training time can be greatly reduced. In other words, low-efficiency devices tend to be selected for training simultaneously, and high-efficiency devices also tend to be selected for training simultaneously. Moreover, for each batch of selected devices, an agreed-upon value of the energy consumption cost is defined as $E_{i,0}$. When the estimated energy consumption cost of a device in the current round of calculation exceeds this agreed-upon value, the excess calculation will be offloaded to the edge computing center for completion. We enhanced the federated averaging algorithm [37] by proposing Federated Averaging with Device Selection and Computing Offload based on time series.

TimeQueue is a time series from small to large. During device selection, a is set as a time-weighted inverse coefficient, and k devices are randomly selected from TimeQueue[rk+ak] in each round.

When $E_{i}>E_{i,0}$, the amount of data reserved for local training is

(11) $$D_{i,comp}=\frac{E_{i,0}}{{\rho \varepsilon \gamma f}_{i}^{2}}$$

Otherwise, it is 0.

When $E_{i}>E_{i,0}$, the amount of data that needs to be offloaded during local training is

(12) $$D_{i,trans}=D_{i}-\frac{E_{i,0}}{{\rho \varepsilon \gamma f}_{i}^{2}}$$

Otherwise, it is 0. The Algorithm 1 is described below.
**Algorithm 1** Federated Averaging with Device Selection and Computing Offload based on time series 1: Server executes:2: initialize M3: **for** each round r = 1, 2, ... **do**4:   **for** each client c in parallel **do**5:     timeQueue ← UpdateTimeQueue(c, timeQueue)6:     S ← SelectDevices(timeQueue, k, α)7:     **for** each client c∈S in parallel **do**8:       $M^{\prime }$ ← ClientUpdate(c, M)9:       $D_{t}$ ← receive $D_{trans}$ from clients10:    **end for**11:    batches ← (data D split into batches of size)12:    **for** each batch b in batches **do**13:      $M^{\prime \prime }$ ← arg min(Loss(M))14:     **end for**15:    M ← WeightedAvg($M^{\prime }$, $M^{\prime \prime }$, $D_{t}$, D)16:  **end for**17:**end for**18:**function** SelectDevices(timeQueue, k, α)19:  r ← (random value in area [0, 1])20:  S ← (random k devices in area timeQueue[rk, rk+αk])21:  return S22:**end function**23:**function** UpdateTimeQueue(c, timeQueue)      ▷Executed on client c14:  time ← (calculate time with Formula (4))25:  Insert(timeQueue, time)26:  return timeQueue27:**end function**28:**function** ClientUpdate(c, M)             ▷Executed on client c29:  energy ← (calculate energy with Formula (5))30:  **if** energy > $E_{0}$ **then**31:    $D_{t}$ ← (calculate $D_{trans}$ with Formula (12))32:    transfer $D_{t}$ to Server33:  **end if**34:  **for** each local epoch i from 1 to E **do**35:    batches ← (data D split into batches of size)36:    **for** each batch b in batches **do**37:      M ← arg min(Loss(M))38:    **end for**39:  **end for**40:  **return** M to Server41:**end function**

Accordingly, the time consumed for data transmission is

(13) $$T_{trans}=\frac{1}{B}\sum_{i=1}^{k}D_{i,trans}$$

where B is the total bandwidth of the system. It should be noted that the data transmission model is significantly simplified, omitting any discussion of queue latency, limitations in MAC protocol performance, the effects of Automatic Repeat request (ARQ) and Forward Error Correction (FEC), and other critical teletraffic engineering considerations. Additionally, bandwidth is allocated to each computing device using average distribution and the formulas give average values. This simplification assumes that devices with lower computational and transmission capabilities will not exceed protocol limitations. Moreover, it assumes the system operates under ideal stability, thereby refraining from accounting for time consumption influenced by factors such as ARQ and FEC, which are challenging to quantify precisely. The same reasoning applies to the following equation.

The energy consumed for data transmission is

(14) $$E_{trans}=\sum_{i=1}^{k}\frac{pkD_{i,trans}}{\sigma B}$$

where p represents the transmission power and σ is a constant coefficient of the transmission rate over the bandwidth.

By using $D_{i,comp}$ in place of $D_{i}$ in Equations (8) and (10), the total training time consumption of the system based on device selection and computation offloading in one round of training can be expressed as

(15) $$T_{total}={T_{comp}+T}_{trans}$$

(16) $$T_{total}=max{\left\{\varepsilon \gamma D_{i,comp}\frac{1}{f_{i}}\right\}}_{i=1}^{k}+\frac{1}{B}\sum_{i=1}^{k}D_{i,trans}$$

Similarly, the total energy consumption is

(17) $$E_{total}=E_{comp}+E_{trans}$$

(18) $$E_{total}=\sum_{i=1}^{k}({\rho \varepsilon \gamma D_{i,comp}f_{i}^{2}}_{i}+\frac{kpD_{i,trans}}{\sigma B})$$

For an edge computing center with strong computing power and a guarantee of sufficient energy, the training time for the typically small amount of offloaded data will be much less than $T_{comp}$, so its energy consumption is not considered.

## 4. Results

As described in this section, we applied the above edge computing framework to construct a system and conducted comparative experiments using the proposed time series-based method of device selection and computation offloading to verify its feasibility as well as its superiority in terms of time and energy costs.

### 4.1. Experimental Setup

As described in this section, the above edge computing framework was applied to construct a system for simulation experiments, which included an edge computing center and 40 edge devices. The number of local training iterations was 10. The average number of CPU clock cycles per unit amount of data was determined by the dataset and the model to be trained. The CPU frequency followed a uniform distribution in the range of [1,2] GHz. The constant capacitance coefficient was set to $10^{-28}$. The total bandwidth was set to 500 MB/s. The transmission power was set to 0.1 W. The constant coefficient of the transmission rate over the bandwidth was set to 0.9. The agreed-upon maximum value of the energy consumption cost was set to 5 J. The number of devices selected in each training round was 15. In these experiments, the time-weighted inverse coefficient was set to different values of 2, 1.6 and 1.2.

In the IIoT domain, various machine learning methods can be applied, including Multilayer perceptrons (MLP), convolutional neural networks (CNN), recurrent neural networks (RNN), support vector machines (SVM), k-nearest neighbor (KNN), Long Short-Term Memory (LSTM), and other deep reinforcement learning networks [53,54,55]. Among these methods, MLP is particularly versatile and adaptable, capable of addressing diverse IIoT tasks such as image processing, data analysis, anomaly detection, and quality control. MLP also offers interpretability, allowing insights into how internal weights and neurons respond to input data. Moreover, its training time scales approximately linearly with the dataset size, aligning with the assumptions in Section 3’s formulas. Therefore, we selected the MLP model for validation in this experiment.

We used the MNIST [56] dataset to verify the effectiveness of the proposed scheme. The MNIST dataset contains 60,000 training data samples and 10,000 test samples. These samples are all 28 × 28 pixel images of handwritten digits. The training samples were divided among the 40 edge devices following a uniform distribution in the range of [1000, 2000]. The MNIST dataset was used to train an MLP model. The Flatten layer converted each 28 × 28 image into a flat vector. Dense layers were fully connected layers: the first Dense layer had 64 neurons with ReLU activation. The output Dense layer had 10 neurons (one for each digit from 0 to 9) with softmax activation for multi-class classification. The model used the Adam optimizer, sparse categorical cross-entropy loss function (suitable for integer-encoded labels like MNIST), and accuracy metric. The model was trained with 5 epochs, a batch size of 32, and a learning rate of 0.001.

In the experiments, we set up a comparative test. In the control group, a random device selection scheme without optimization was applied as a baseline against which to compare the time series-based scheme for device selection and computation offloading proposed in this paper. First, the convergence and accuracy of the proposed scheme were verified on the training and test datasets and compared with those of the control group. Then, different time-weighted inverse coefficients were set to evaluate the relationship between the training time and energy consumption for further comparison of the proposed scheme and the control scheme.

### 4.2. Analysis of Results

In this section, the effectiveness of the proposed scheme is evaluated in terms of model convergence and accuracy, and its superiority in terms of training time and energy consumption is investigated in comparison with the control scheme.

#### 4.2.1. Model Loss and Accuracy

**Figure 4:**
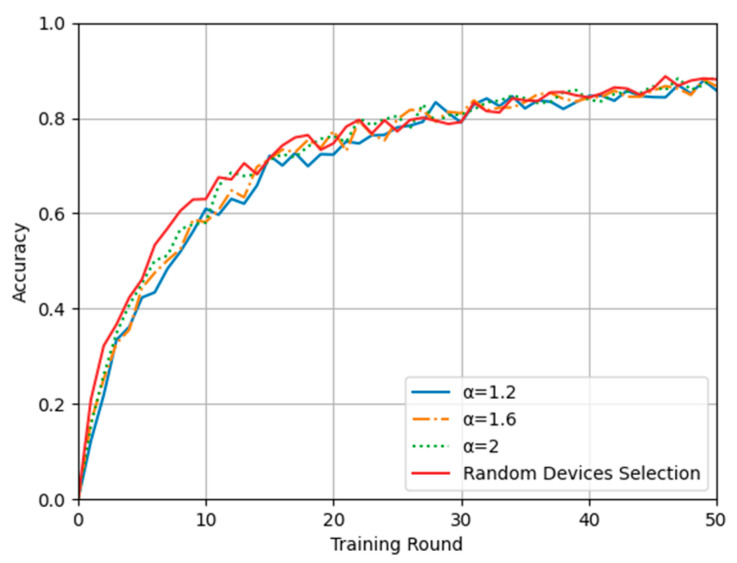
Plot of training accuracy.

**Figure 5:**
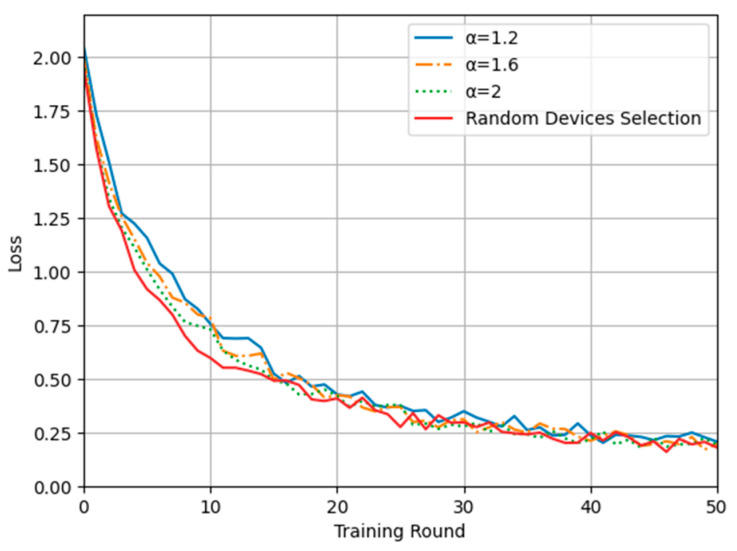
Plot of training loss.

In this experiment, the training performance and model convergence ability of the federated learning algorithm based on the proposed time series-based device selection and computation offloading method are compared with those of the traditional federated learning algorithm with random device selection. Figure 4 and Figure 5 show plots of the model accuracy and loss, respectively, during training using the random device selection algorithm and the proposed algorithm with different time-weighted inverse coefficients. It can be seen from the comparative analysis that when the number of training rounds is less than 20, the model accuracy and convergence speed achieved with the proposed scheme show small differences compared with those achieved with random device selection, and the smaller the time-weighted inverse coefficient is, the larger the difference is. When the number of training rounds is more than 20, however, there is no large gap between the model convergence and accuracy with the different schemes. Although the difference is small, it is seen that because the proposed device selection strategy preferentially selects training batches consisting of similar devices, it results in greater sample similarity and consistency than the completely random selection method when the number of training rounds is small. However, as the number of training rounds increases, all devices gradually come to be treated equally in long-term device selection, so the data homogeneity is eliminated, and the model accuracy and convergence results are not affected. Therefore, these results show that the proposed method may have a small impact on model convergence and accuracy in the case when less training time is available, and the smaller the time-weighted inverse coefficient is, the greater the impact; however, it does not affect the actual final model convergence and accuracy.

#### 4.2.2. Curve of Training Time over Multiple Training Rounds

**Figure 6:**
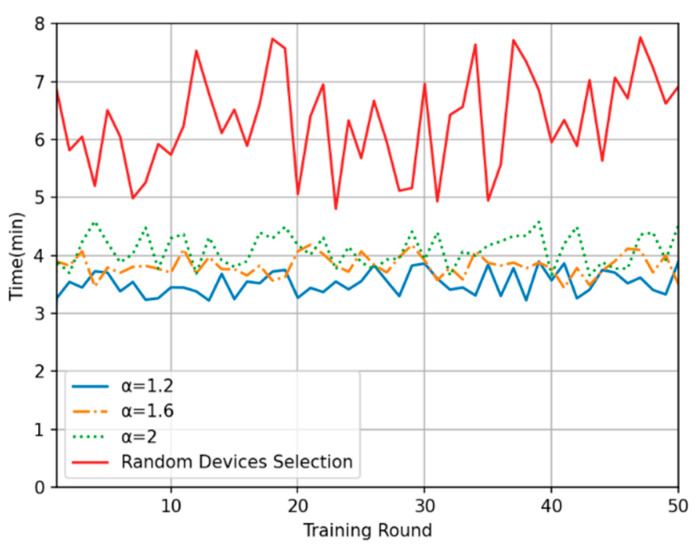
Plot of single-round training times.

**Figure 7:**
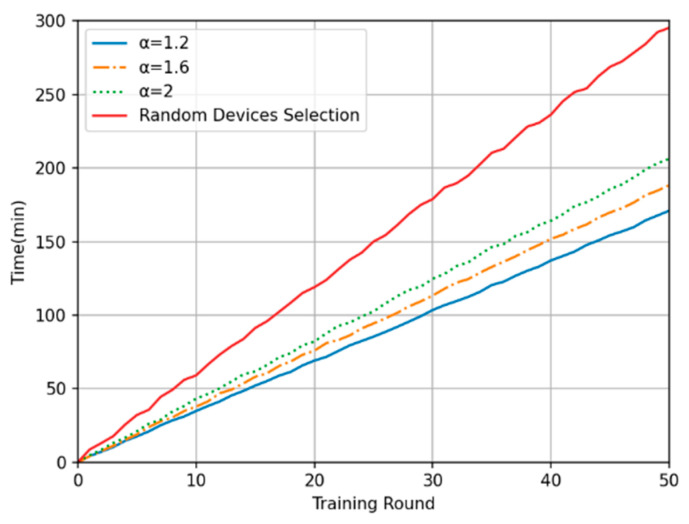
Plot of cumulative training time.

In this experiment, the training time in each round and the cumulative training time were compared between the proposed method and the random device selection method. Figure 6 shows the overall time consumption in each of the 50 training rounds. The model training time with the proposed method is generally 30% to 50% less than that with the random device selection method, and the smaller the time-weighted inverse coefficient is, the shorter the average training time in each round. Moreover, regarding the oscillation amplitude of the curve, the random device selection method results in the largest oscillations, while the oscillation amplitude under the proposed method is slightly reduced with a decrease in the time-weighted inverse coefficient. The relevant experiments in reference [29] demonstrate that when the client data participating in each training round cannot cover all data distributions, selecting more clients to participate can accelerate the convergence speed of the federated model. Therefore, Figure 4 does not show any superiority in terms of training rounds. However, as the time-weighted inverse coefficient decreases, the system tends to select a more centralized data scale and offloads data exceeding the threshold to the edge computing center for computation, thereby reducing processing time. Figure 7 shows the total time consumption of the system after 50 rounds of training with each method. It shows that the proposed time series-based method of device selection and computation offloading can reduce the total training time to a certain extent compared with the random device selection method over the same number of training rounds. These experimental results verify the superiority of the proposed method in terms of time consumption.

#### 4.2.3. Curve of Energy Consumption over Multiple Training Rounds

**Figure 8:**
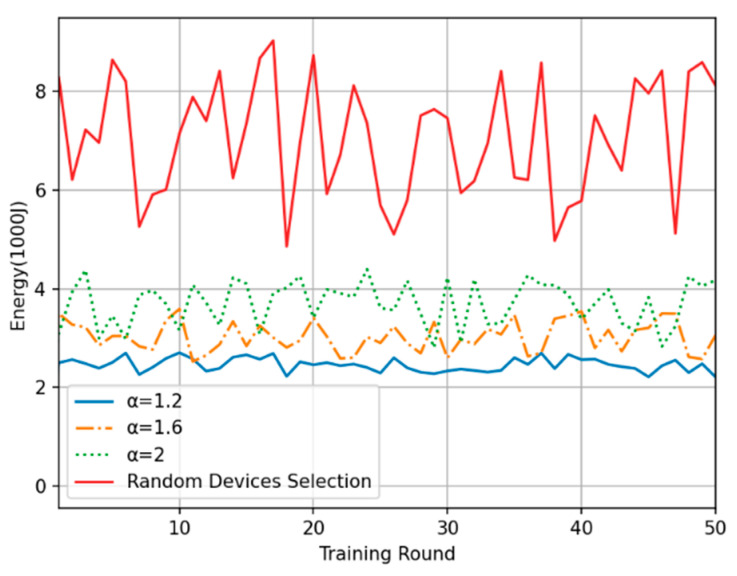
Plot of single-round energy consumption.

**Figure 9:**
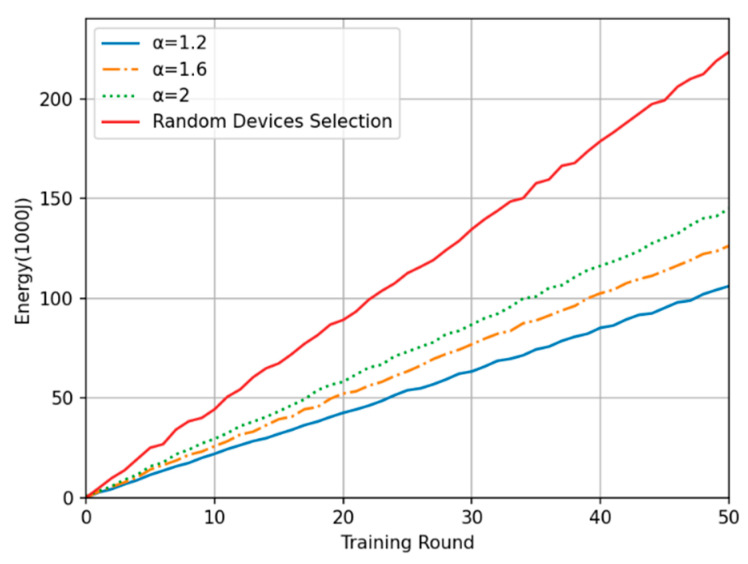
Plot of cumulative energy consumption.

In this experiment, the energy consumption per training round and the cumulative training energy consumption (estimated according to Equation (18) in Section 3.3 and excluding the energy consumption of the edge computing center) were compared between the proposed method and the random device selection method. Figure 8 shows the overall energy consumption in each of the 50 training rounds. The training energy consumption under the proposed method is generally 35% to 55% less than that under the random device selection method, and the smaller the time-weighted inverse coefficient is, the lower the average training energy consumption in each round. Moreover, regarding the oscillation amplitude of the energy consumption curve, the random device selection method results in the largest oscillations, while the oscillation amplitude under the proposed method is slightly reduced with a decrease in the time-weighted inverse coefficient. These findings are consistent with the analysis presented in the previous subsection. Figure 9 shows the total energy consumption of the system after 50 rounds of training with each method. It shows that the proposed time series-based method of device selection and computation offloading can somewhat reduce the total training energy consumption on the device side compared with the random device selection method over the same number of training rounds. These experimental results verify the superiority of the proposed method in terms of time consumption.

#### 4.2.4. Comparison with Related Methods

As shown in Table 1, our method is compared with other related methods, including FedAvg [29], DCS [40], FedCS [41], FedMCCS [42], and FedCCPS [43], in terms of feature collection, optimization goals, strategies, and time improvements compared to a random device selection method. It should be noted that due to differences in feature collection and optimization goals, direct horizontal comparisons of these methods on the same dimension are relatively challenging. However, compared to the random device selection method, our approach demonstrates superiority over other methods in scenarios involving more extensive feature collection, applying the time series-based method of device selection and computation offloading. 

## 5. Discussion

This paper proposes a three-layer, software-defined AI-oriented edge computing framework for the IIoT, which can overcome the problem of the high hardware and software coupling of edge devices in the IIoT as well as the difficulties of deploying and delivering AI models and interacting with the device side to some extent. Based on this framework, the design and implementation of an AI-oriented edge computing system are also presented. In the proposed architecture, the edge receives AI models deployed from the cloud for training, and model training is performed at the edge based on on-site data. This method of training at the edge supports a variety of IIoT transmission protocols and facilitates access to various common IIoT control devices and sensor devices to meet the needs of different application scenarios. To a large extent, this approach also protects the privacy and security of data, in accordance with the concepts of big data AI, model sharing and data privacy. In addition, to reduce the delay of model training and the energy consumption of the devices while adapting to the needs of less efficient nodes, this paper proposes a time series-based method of device selection and computation offloading. Experiments verify the feasibility of this method compared with traditional random device selection in terms of training accuracy and model convergence as well as its superiority in terms of time consumption and energy consumption.

However, there are still some shortcomings and limitations in this study. In the experiments, we found that the device access system based on container virtualization has a certain probability of exhibiting a small amount of uncertain jitter in response. This jitter presents a great challenge for industrial application scenarios that require high real-time performance.

In future work, we will focus on improving the real-time performance of the system, including increasing the efficiency of data transmission, increasing the effectiveness of the time-sharing scheduling strategy, and optimizing the computing resource allocation method. The overall framework of IIoT edge computing for AI models is currently undergoing constant development, and we hope that the architecture proposed in this paper can serve as a reference for researchers in this area.

## Figures and Tables

**Table 1:** Comparison with related methods.

Methods	Feature Collection	Optimization Goals	Strategies	Time Improvements Compared to Random Device Selection Method
FedAvg	-	-	Random device selection method	-
DCS	Device communication time	Minimizing overall communication cost	Client selection threshold filtering	32.67%
FedCS	Local model training time; model transmission time	Maximizing the number of device selections	Greedy algorithm under the knapsack constraint problem	-
FedMCCS	CPU frequency; Memory; device Energy	Maximizing the number of device selections	Double-layer greedy algorithm under the knapsack constraint problem	-
FedCCPS	CPU frequency; size of dataset; transmission power	Minimizing overall training time	Federated client cluster and latency-prediction selection	21%
Ours	CPU frequency; size of dataset; transmission power; bandwidth	Minimizing overall training time within limits of energy consumption	Time series-based method of device selection and computation offloading	30–50%

## Data Availability

The MNIST dataset used in the experimental section is sourced from: Deng, L. The MNIST database of handwritten digit images for machine learning research. IEEE Signal Processing Magazine, 29(6), 141–142, 2012. It can be downloaded from https://yann.lecun.com/exdb/mnist/ (accessed on 11 November 2022).
